# Online clinical pathway for chronic kidney disease management in primary care: a retrospective cohort study

**DOI:** 10.1186/s12882-021-02533-5

**Published:** 2021-10-06

**Authors:** Maoliosa Donald, Michelle D. Smekal, Meghan J. Elliott, Kerry McBrien, Robert G. Weaver, Braden J. Manns, Marcello Tonelli, Aminu Bello, Sharon E. Straus, Nairne Scott-Douglas, Kailash Jindal, Brenda R. Hemmelgarn

**Affiliations:** 1 Department of Medicine, University of Calgary, 3330 Hospital Drive NW, Calgary, AB T2N 4N1 Canada; 2 Department of Family Medicine, Cumming School of Medicine, University of Calgary, 3330 Hospital Drive NW, Calgary, AB T2N 4N1 Canada; 3 Faculty of Medicine & Dentistry, University of Alberta, 2J2.01 Walter C MacKenzie Health Sciences Centre, Clinical Sciences Building, 8440 112 St NW, Edmonton, AB T6G 2B7 Canada; 4 Department of Family & Community Medicine, University of Toronto, Toronto, ON Canada

**Keywords:** Chronic kidney disease, Primary care, Clinical pathway, Quality improvement, Knowledge translation

## Abstract

**Background:**

Clinical pathways aim to improve patient care. We sought to determine whether an online chronic kidney disease (CKD) clinical pathway was associated with improvements in CKD management.

**Methods:**

We conducted a retrospective pre/post population-based cohort study using linked health data from Alberta, Canada. We included adults 18 years or older with mean estimated glomerular filtration rate (eGFR) < 60 ml/min/1.73m^2^. The primary outcome was measurement of an outpatient urine albumin creatinine ratio (ACR) in a 28-day period, among people without a test in the prior year. Secondary outcomes included use of guideline-recommended drug therapies (angiotensin-converting enzyme inhibitors, angiotensin receptor blockers and statins).

**Results:**

The study period spanned October 2010 to March 2017. There were 84 independent 28-day periods (53 pre, 31 post pathway implementation) including 345,058 adults. The population was predominantly female (56%) with median age 77 years; most had category 3A CKD (67%) and hypertension (82%). In adjusted segmented regression models, the increase in the rate of change of ACR testing was greatest in Calgary zone (adjusted OR 1.19 per year, 95% CI 1.16–1.21), where dissemination of the pathway was strongest; this increase was more pronounced in those without diabetes (adjusted OR 1.25 per year, 95% CI 1.21–1.29). Small improvements in guideline-concordant medication use were also observed.

**Conclusions:**

Following implementation of an online CKD clinical pathway, improvements in ACR testing were evident in regions where the pathway was most actively used, particularly among individuals without diabetes.

**Supplementary Information:**

The online version contains supplementary material available at 10.1186/s12882-021-02533-5.

## Background

Chronic kidney disease (CKD) affects approximately 11% of adults in Canada and is associated with considerable morbidity, mortality, and health care costs [1]. Early intervention, including targeted testing for albuminuria and medical management with angiotensin-converting enzyme inhibitors (ACEi), angiotensin receptor blockers (ARB) and statins, where indicated, can delay CKD progression and/or reduce cardiovascular risk [2–6]. Despite availability of effective therapies, important care gaps are evident [7, 8], with over 80% of Canadian adults with CKD not tested for albuminuria and approximately 50% of older adults with CKD not prescribed guideline-recommended medications [9, 10]. Since over 90% of patients with CKD are managed in primary care [11], and primary care providers have indicated the need for concise point-of-care guidelines [12], an online CKD clinical pathway (www.ckdpathway.ca) was developed to support guideline-concordant care in community settings [13, 14].

Clinical pathways are effective tools to streamline clinical guidelines and improve efficiency of care, and have become increasingly common, particularly in hospital settings [15, 16]. Despite widespread implementation, the effectiveness of clinical pathways targeted toward community practice settings has not been well studied [15, 17]. Furthermore, while several pathways have targeted aspects of CKD identification and/or management, the majority of these are static, may not be updated to incorporate current guidelines, and have not undergone rigorous evaluation [17]. We aimed to determine whether implementation of an online CKD clinical pathway was associated with improvements in CKD management, including targeted urine albumin-creatinine ratio (ACR) testing and guideline-recommended drug therapy (ACEi, ARB and statins) [2].

## Methods

**Fig. 1:**
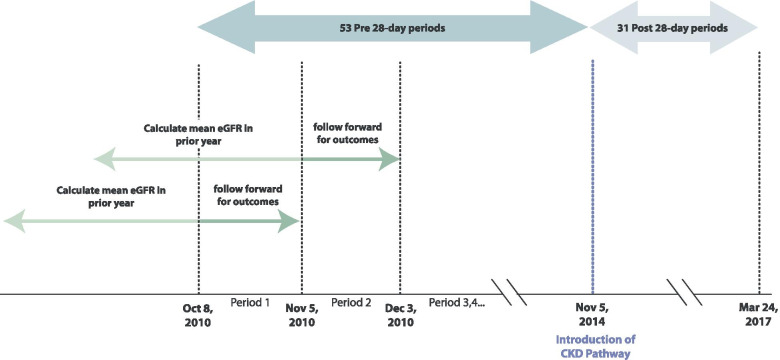
Study design

We conducted a retrospective population-based pre-post cohort study using linked administrative health and laboratory data from Alberta, Canada [18] (Fig. 1). The CKD pathway was launched November 5, 2014. The pre-intervention period comprised 53 28-day periods from October 8, 2010 to November 4, 2014, and the post period comprised 31 28-day periods from November 5, 2014 to March 24, 2017. Each 28-day period included Alberta residents aged 18 years or older with mean estimated glomerular filtration rate (eGFR) < 60 ml/min/1.73m^2^, using all outpatient serum creatinine measurements in the prior year. We excluded people who had commenced dialysis or received a kidney transplant prior to the last day of the 28-day period. We chose 28-day periods rather than months given variability in laboratory testing by day of week, and variability in the number of weekdays in a calendar month year to year. Years were divided into thirteen 28-day periods, with the extra day (two in leap years) assigned to the final period. All methods were carried out in accordance with relevant guidelines and regulations.

### The online CKD pathway

The CKD pathway was developed by a multidisciplinary group of stakeholders to provide access to concise point-of-care guidelines for the identification, medical management, and referral of adults with CKD [13]. The pathway incorporated relevant clinical practice guidelines [19–24]; results of the rigorous pre-implementation development and testing process were published previously [13]. The primary goals of the pathway were to improve early identification (through targeted ACR testing of high-risk patients) and appropriate medical management (prescription of ACEi/ARB and/or statin, where indicated). Although the CKD pathway is freely available (www.ckdpathway.ca), implementation was targeted to primary care providers in Alberta. A multifaceted strategy was used to support implementation [25]. Website analytics showed that pathway uptake was consistently greatest in Calgary, Alberta, with nearly three times as many users (31%) compared to Edmonton (11%), a similar-sized city in Alberta [25].

### Outcomes

The primary outcome was assessment of albuminuria as defined by an outpatient random urine ACR measurement, in a 28-day period, among people without a test in the prior year (to reflect new assessment of albuminuria). Secondary outcomes were related to CKD management and included use of an ACEi/ARB or statin, defined as dispensation of a prescription during a 28-day period. ACEi/ARB use was assessed in individuals with diabetes as well as in those without diabetes but whose most recent ACR was > 300 mg/g (30 mg/mmol), indicating severe (A3) albuminuria. Statin use was assessed in individuals with diabetes, and in those without diabetes who were 50 years of age or older (as per guideline recommendations [19]). All outcomes were treated as binary.

### Measurement of covariates

Diabetes, hypertension and other comorbidities were identified from physician claims and hospital discharge records using validated algorithms [26–28]. Albuminuria was defined using ACR and categorized as A1 (< 30 mg/g), A2 (30–300 mg/g) and A3 (> 300 mg/g). We determined dates of kidney transplants and dialysis initiation by linking to provincial dialysis and transplant registry data. We categorized patients using geographically defined health zones in Alberta into three categories (Calgary, Edmonton, Other) based on postal code of the person’s residence (Fig. S1). We also linked aggregated 2011 and 2016 federal census data to obtain neighbourhood income quintile and urban/rural location. Calgary and Edmonton zones each include a large metropolitan centre (> 1 million residents) and smaller neighbouring towns. The Other category includes zones in South, North, and Central Alberta, which are primarily rural with 1–2 smaller cities in each region (< 100,000 residents). We defined all covariates at the beginning of each 28-day period.

### Statistical analysis

We determined the association between CKD pathway implementation and outcomes using segmented regression analysis. We used logistic regression within a generalized estimating equation framework to account for individuals’ repetition across periods, with an exchangeable covariance matrix and with robust standard errors that are insensitive to misspecification of the correlation matrix. We adjusted models for covariates that were chosen a priori [age, sex, eGFR category, albuminuria category (where relevant), comorbidities, neighbourhood income quintile, rural/urban residence and the 13 periods within the year, to account for seasonality]. We included a term for the rate of change of the outcome in the pre period (slope, expressed as an odds ratio per year) and modeled the effect of the intervention by including a term for the change in slope between pre and post periods, also expressed as an odds ratio per year. We did not include a term for a sudden increase in referrals, based on our understanding of the way the intervention would affect the outcome, which is key to determining how to model the effects of an intervention [29]. Specifically, we thought that physicians would gradually start referring more patients for ACR testing as awareness of the CKD Pathway increased.

Given differences in pathway use trends [25], we assumed uptake of the CKD pathway varied across Alberta. We tested for effect modification by health zone (Calgary, Edmonton, and Other zones) by including terms for zone and interactions with pre-intervention slope and pre-post change in slope. If the interaction terms between zone and pre-post change in slope were statistically significant, results were reported by zone.

In sensitivity analyses for the primary outcome, we restricted the cohort to people whose mean eGFR was calculated from at least two serum creatinine measurements in the prior year, to reduce potential misclassification of CKD. In other sensitivity analyses, we analyzed the prescription drug outcomes using a modified calendar quarter (three quarters comprising three 28-day periods and one quarter comprising four 28-day periods) rather than a 28-day period, because a 28-day period captures only a portion of prescriptions for an individual. Most individuals were prescribed an ACEi, ARB or statin for a 30-, 90- or 100-day period.

All statistical analyses were conducted in Stata 16 (StataCorp, College Station, TX). The study was approved by the Health Research Ethics Board of the University of Calgary.

## Results

**Fig. 2:**
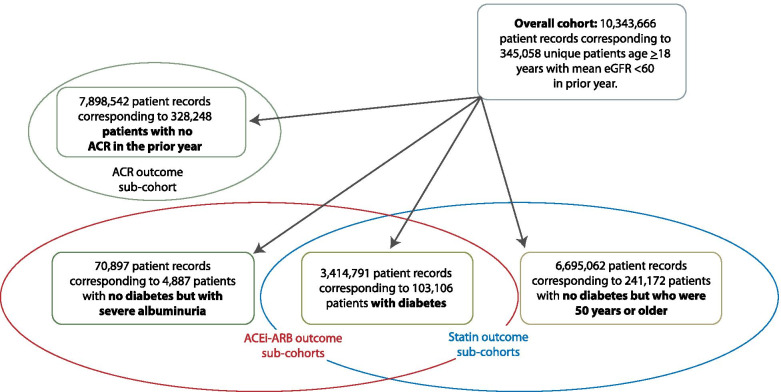
Cohort diagram

The 84 independent 28-day periods included 10,343,666 patient records corresponding to 345,058 unique patients. Characteristics of the overall cohort and the sub-cohort used for analysis of the primary outcome (i.e., those without a urine ACR in the prior year) are provided in Table 1 and Fig. 2. The overall cohort was predominantly female (56.4%), with median age 77 years. The majority had category 3A CKD (66.8%), with a high prevalence of hypertension (82.2%) and diabetes (33.0%). For analysis of secondary outcomes, the sub-cohort of people with diabetes was almost 50-fold larger than the sub-cohort of people with severe albuminuria and no diabetes (Table S1). Compared to people with severe albuminuria and no diabetes, people in the diabetes cohort were older (median age 76.5 years), more likely to be female (50.6%) and to have an eGFR > 45 ml/min/1.73m^2^ (57.3%). People in the sub-cohort who were older than 50 years and without diabetes were more likely to be female (59.5%) with a lower prevalence of comorbidities than people with diabetes.

**Table 1:** Characteristics of the overall cohort, and the sub-cohort eligible for the primary outcome (no ACR measurement in the prior year), (% unless otherwise noted)

Characteristic	Overall cohort (*N* = 10,343,666 patient records)	Sub-cohort eligible for the primary outcome (*N* = 7,898,542 patient records)
Number of unique patients	345,058	328,248
Number of times each patient appears in the cohort, median (IQR)	22 (11,46)	16 (8,36)
Age in years, median (IQR)	76.8 (68.2, 84.1)	77.7 (68.7, 84.9)
Female	56.4	58.4
Health zone		
Calgary Zone	34.9	36.4
Edmonton Zone	29.9	27.7
Other zones	35.2	35.9
Patient records with an ACR measurement	3.05	1.39
Most recent ACR in past year		
Normal/mild (A1: < 30 mg/g)	13.5	–
Moderate (A2: 30–300 mg/g)	6.8	–
Severe (A3: > 300 mg/g)	3.3	–
Unmeasured	76.4	100
eGFR category (ml/min/1.73m^2^)		
3a (45–59)	66.8	68.7
3b (30–44)	25.1	24.0
4 (15–29)	7.2	6.5
5 (< 15)	0.9	0.9
Number of outpatient serum creatinine measurements in past year		
1	45.0	50.4
2–3	35.2	33.1
≥4	19.8	16.5
Alcohol misuse	3.2	3.2
Asthma	4.8	4.7
Atrial fibrillation	17.7	18.4
Cancer	13.7	14.2
Chronic heart failure	21.2	21.5
Chronic pulmonary disease	26.5	26.8
Chronic viral hepatitis B	0.1	0.1
Cirrhosis	0.7	0.7
Dementia	11.5	13.2
Diabetes	33.0	22.8
Epilepsy	2.3	2.4
Hypertension	82.2	79.8
Hypothyroidism	23.1	23.7
Inflammatory bowel disease	1.8	1.9
Irritable bowel syndrome	3.2	3.3
Multiple sclerosis	0.7	0.8
Metastatic cancer	4.0	4.3
Myocardial infarction	8.3	8.0
Parkinson’s disease	2.1	2.3
Peripheral vascular disease	5.3	5.2
Psoriasis	1.3	1.3
Rheumatoid arthritis	5.9	6.2
Schizophrenia	1.4	1.5
Stroke or TIA	20.8	21.2
Neighbourhood income quintile		
1 (lowest)	25.4	25.1
2	22.9	22.8
3	19.7	19.8
4	16.0	16.0
5 (highest)	15.9	16.2
Unknown	0.1	0.1
Rural residence		
Urban	77.7	77.0
Rural	22.3	22.9
Unknown	0.1	0.1

*IQR* Interquartile range, *ACR* Urine albumin/creatinine ratio, *eGFR* Estimated glomerular filtration rate, *TIA* Transient ischemic attack

### Primary outcome: pre-post change in ACR measurement

**Fig. 3:**
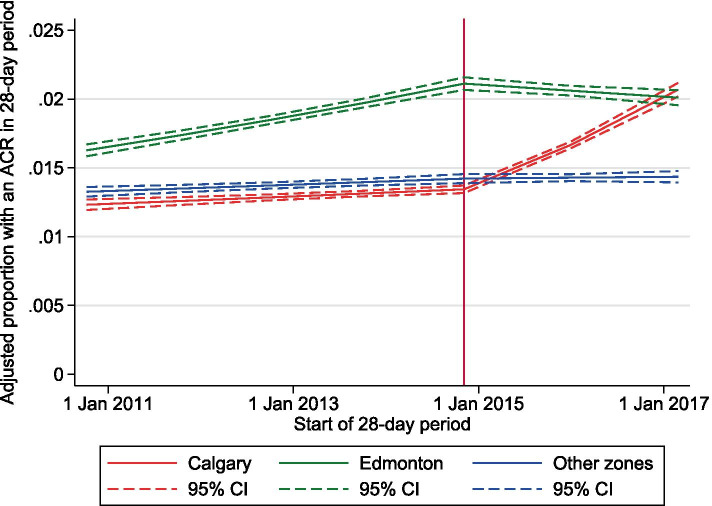
Segmented regression model for ACR measurement (primary outcome), by zone, including 95% confidence intervals (dashed lines). Adjusted for age, sex, eGFR category, the thirteen 28-day periods, neighbourhood income quintile, rural residence, and all comorbidities in Table 1. Being logistic models, they are linear on the log(odds) scale, but are also very nearly linear on the probability scale

Overall the percentage of people with an ACR test in a 28-day period, among those who had not had a measurement in the prior year, was 1.31% in the pre period and 1.51% in the post period. The increase was greatest in the Calgary (1.17 to 1.50%), followed by Edmonton (1.57 to 1.80%) and Other zones (1.22 to 1.30%). In adjusted segmented regression models, interactions between zone and pre-post change in slope for ACR measurement were significant (all *P* < 0.001), so we calculated zone-specific estimates (Table 2; Fig. 3). In the Calgary zone there was a statistically significant pre-post increase in slope (adjusted OR 1.19 per year, 95% CI 1.16–1.21), while in the Edmonton zone there was a significant pre-post decrease in slope (adjusted OR 0.92 per year, 95% CI 0.89–0.94). Prior to CKD pathway implementation, there was a substantially higher rate of ACR testing in Edmonton zone, which was increasing over time; the decrease in slope following CKD pathway implementation was associated with a stable/slow decline in the rate of ACR testing post-intervention in that zone (Fig. 3). In Other zones, there was little pre-post change in slope.

**Table 2:** Estimates of the rate of change in ACR measurement in the pre period and the post period, and the pre-to-post change (by zone; in the full cohort and stratified by diabetes)

Cohort	Patient records	Zone	Odds ratios for rate of change per year in ACR measurement		
			Pre period OR (95% CI)	Post period OR (95% CI)	Pre to post change OR (95% CI) (outcome)
Full	7,898,542	Calgary	1.02 (1.01–1.03)	1.21 (1.19–1.23)	1.19 (1.16–1.21)
		Edmonton	1.07 (1.06–1.08)	0.98 (0.96–0.99)	0.92 (0.89–0.94)
		Other	1.02 (1.01–1.03)	1.00 (0.99–1.02)	0.99 (0.96–1.01)
Patients with diabetes	1,798,159	Calgary	1.01 (0.99–1.02)	1.10 (1.07–1.12)	1.09 (1.06–1.12)
		Edmonton	1.03 (1.02–1.04)	0.94 (0.92–0.97)	0.92 (0.89–0.95)
		Other	1.00 (0.99–1.02)	0.99 (0.97–1.01)	0.98 (0.95–1.01)
Patients without diabetes	6,100,383	Calgary	1.04 (1.03–1.06)	1.30 (1.28–1.33)	1.25 (1.21–1.29)
		Edmonton	1.12 (1.11–1.14)	1.01 (0.98–1.03)	0.90 (0.87–0.93)
		Other	1.04 (1.03–1.06)	1.02 (0.99–1.04)	0.98 (0.94–1.01)

All P for interactions between zone and the primary outcome were < .001. Adjusted for age, sex, eGFR category, the thirteen 28-day periods, neighbourhood income quintile, rural residence, and all comorbidities in Table 1

**Fig. 4:**
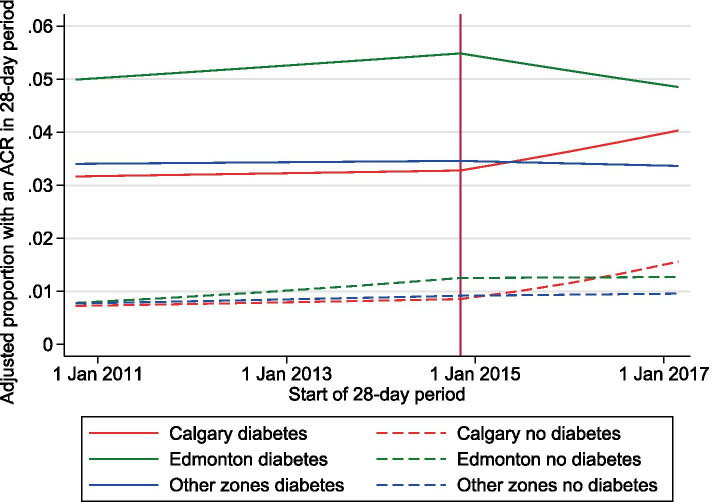
Segmented regression models for ACR measurement (primary outcome), by zone, among those with and without diabetes. Adjusted for age, sex, eGFR category, the thirteen 28-day periods, neighbourhood income quintile, rural residence, and all comorbidities in Table 1. Being logistic models, they are linear on the log(odds) scale, but are also very nearly linear on the probability scale

Significant interactions between zone and pre-post change were also evident when we stratified by diabetes (all P for interaction < 0.001) (Fig. 4). Overall, the prevalence of ACR testing was higher among those with (3.1%) than those without diabetes (0.9%). Again, there were significant pre-post increases in slope in Calgary zone, significant decreases in slope in Edmonton zone, and little change in Other zones, among those with and without diabetes. The increase in Calgary zone was greater among those without diabetes (adjusted OR 1.25 per year, 95% CI 1.21–1.29) than those with diabetes (adjusted OR 1.09 per year, 95% CI 1.06–1.12).

The pre-post change in slope in Calgary zone corresponded to approximately 800 (95% CI 700–900) additional people tested in the first year, and 2400 (95% CI 2100–2700) in the second year (cohort size 43,000). Of the additional people tested, about three quarters were without diabetes.

Because the segmented regression models were constrained to a change in slope at the time of CKD pathway implementation, we also compared the proportions predicted by the models for each zone against the adjusted proportions with an ACR measurement in each 28-day period from a model with the 84 independent periods as a categorical variable (Figs. S2 and S1). While the adjusted proportions for Calgary zone are consistent with the results from the segmented regression (Fig. S2), the adjusted proportions for Edmonton zone showed a decrease in ACR testing approximately 2 years after CKD pathway implementation, rather than around the time of the intervention (Fig. S1).

The results of the sensitivity analyses for the primary outcome (sub-cohort limited to those with 2 or more serum creatinine measurements in the prior year) were broadly similar to the main analyses (Table S2).

### Secondary outcomes

#### Pre-post change in ACEi/ARB use

The prevalence of ACEi/ARB use (defined as dispensation of a prescription in a 28-day period) during the study period was 30.4% in the diabetes cohort and 25.8% in the albuminuria cohort. The interaction terms between zone and pre-post change in slope were significant in both cohorts. In the diabetes cohort there was a small but statistically significant pre-post increase in slope in Calgary zone (adjusted OR 1.02 per year, 95% CI 1.00–1.04) and Edmonton zone (adjusted OR 1.02 per year, 95% CI 1.01–1.04) but not in Other zones (Table 3, Fig. S4). In the albuminuria cohort, the pre-post change in slope was not significant in any of the zones (Fig. S5). The pre-post increase in the diabetes cohort represent an additional 55 (95% CI 9–104) people in Calgary Zone (cohort size 14,600) and 58 (95% CI 18–101) people in Edmonton Zone (cohort size 12,500) dispensed an ACEi/ARB per 28-day period over a one-year timeframe.

**Table 3:** Estimates of the pre-post change in slope for ACEi/ARB use in a 28-day period: diabetes cohort and no diabetes/severe albuminuria cohorts

	Diabetes (*N* = 3,414,791 patient records)		No diabetes, A3 albuminuria (*N* = 70,897 patient records)	
Zone	P for interaction	OR (95% CI) (per year)	P for interaction	OR (95% CI) (per year)
Overall	–		–	
Calgary Zone	Ref	1.02 (1.00–1.04)	Ref	0.91 (0.83–1.00)
Edmonton Zone	0.72	1.02 (1.01–1.04)	0.05	1.04 (0.95–1.14)
Other zones	0.02	0.99 (0.98–1.01)	0.04	1.07 (0.95–1.20)

Adjusted for age, sex, eGFR category, ACR category (diabetes cohort only), the thirteen 28-day periods, neighbourhood income quintile, rural residence, and all comorbidities in Table 1

In sensitivity analyses, the overall prevalence of ACEi/ARB use in a modified calendar quarter during the study period was 58% in the diabetes cohort and 55% in the no diabetes/albuminuria cohort. In the diabetes cohort, the interactions between zone and pre-post change in the outcome were not significant, and there was a small but statistically significant overall pre-post increase in the outcome (adjusted OR = 1.03 per year, 95% CI 1.02–1.04). In the albuminuria cohort, the interactions were significant. Calgary zone showed a significant decrease from pre to post (adjusted OR = 0.84 per year, 95% CI 0.73–0.96), while the change was not significant in the other two regions (Table S3).

#### Pre-post change in statin use

The overall prevalence of statin use in a 28-day period during the study period was 12.9% in the no diabetes/older than age 50 cohort and 25.4% in the diabetes cohort. The interaction terms between zone and pre-post change in slope were significant in both cohorts (Table 4, Figs. S6, S7). In analysis by zone, there was a small but statistically significant pre-post increase in slope for statin use for Calgary and Edmonton zones but not in Other zones. In both cohorts, the increase was larger in Edmonton zone than in Calgary zone. In the diabetes cohort, the pre-post increase represents an additional 75 people (95% CI 27–118) in Calgary Zone (cohort size 14,600) and 127 people (95% CI 87–167) in Edmonton zone (cohort size 12,500) who were dispensed a statin per 28-day period, over a one-year timeframe. In the no diabetes/over 50 cohort, it represents an additional 58 people (95% CI 4–112) in Calgary Zone (cohort size 37,000) and 89 people (95% CI 54–125) in Edmonton Zone (cohort size 19,500) who were dispensed a statin per 28-day period, over a one-year timeframe.

**Table 4:** Estimates of the pre-post change in slope for statin use in a 28-day period, for the diabetes cohort and the no diabetes/over 50 years old cohort

	Diabetes (N = 3,414,791 patient records)		No diabetes, older than 50 (*N* = 6,895,062 patient records)	
Zone	P for interaction	OR (95% CI) (per year)	P for interaction	OR (95% CI) (per year)
Calgary Zone	Ref	1.03 (1.01–1.05)	Ref	1.02 (1.00–1.03)
Edmonton Zone	0.03	1.06 (1.04–1.08)	0.02	1.04 (1.03–1.06)
Other zones	0.15	1.01 (0.99–1.03)	0.17	1.00 (0.99–1.02)

Adjusted for age, sex, eGFR category, ACR category, the thirteen 28-day periods, neighbourhood income quintile, rural residence, and all comorbidities in Table 1

The prevalence of statin use in a modified calendar quarter during the study period was 48% in the diabetes cohort and 27% in the no diabetes/older than age 50 cohort. In sensitivity analyses, we again found that zone interactions were significant in both cohorts. In both cohorts, there was a significant pre-post increase in slope in all 3 zones, with Edmonton zone showing a slightly larger increase (adjusted OR 1.09 per year, 95% CI 1.07–1.11 in the diabetes cohort; adjusted OR 1.06 per year, 95% CI 1.05–1.08 in the no diabetes/older than 50 cohort; Table S4).

## Discussion

We conducted a population-based cohort study to evaluate implementation of an online CKD clinical pathway targeted to primary care providers. In this cohort of 345,058 adults with CKD we found small but statistically significant higher rates of ACR testing in Calgary zone following CKD pathway implementation, particularly among people without diabetes. Small improvements in guideline-recommended pharmacological therapy (ACEi/ARB and statins) were observed in both Calgary and Edmonton zones in most cohorts. While the observed improvements were small, at a population-based level they may have considerable benefit and the pathway itself required minimal resources and time to maintain once established.

Uptake of the CKD pathway was greater in Calgary zone compared to other zones [25], and may have contributed to the observed increase in ACR testing in that zone. Active dissemination, such as small group education sessions, is a known contributor to uptake of new knowledge amongst physicians; additional dissemination efforts, particularly those that involved outreach visits and were targeted to the needs of the local context, may have improved CKD pathway uptake [30, 31]. There were no significant pre-post changes evident in Other zones (South, Central and North), which encompass the less-populated regions of Alberta. This could be related to fewer dissemination opportunities and the unique challenges experienced treating patients in rural locations, such as access to care barriers and WiFi access and reliability issues [10, 32]. In Edmonton Zone, ACR testing increased steadily in the Pre period and for almost 2 years into the Post period. The relative decrease that occurred at that time may therefore have been unrelated to the CKD pathway implementation.

We also observed a greater increase in ACR testing among individuals without diabetes in the Calgary zone following CKD pathway implementation. Research demonstrates a gap in guideline-concordant CKD care for individuals without diabetes [33, 34]. Despite an increase in ACR testing following implementation, most of these individuals still do not undergo ACR testing, indicating a persisting gap in the application of knowledge regarding management of CKD. Additional dissemination efforts may help to close this gap; however, care for patients with CKD is complex. Patients often have multiple comorbidities, and studies suggest that providers may receive conflicting messages from the various prevention and treatment guidelines [35]. Moreover, while clinical pathways providing streamlined recommendations at the point-of-care may improve guideline-concordant treatment, the workload and time constraints faced by primary care practitioners remains a significant barrier to achieving optimal care [10, 35–38].

Despite a small but statistically significant improvement in ACR testing in Calgary zone, we did not observe a meaningful improvement in guideline-concordant medical management (ACEi/ARBs and statin prescribing). While early diagnosis is critical, appropriate medical treatment is necessary to slow disease progression. Studies have similarly found low rates of CKD recognition despite high rates of screening [39–42] and targeted intervention to improve CKD awareness and treatment [43–45], indicating that CKD awareness alone may not be sufficient to improve appropriate medical management.

Although several clinical pathways targeting CKD have been developed and evaluated [17], they mainly focus on review of initial pathway development and implementation [13, 46, 47]. While pathways have been found to improve clinical outcomes and reduce complications, particularly for invasive interventions [15], we are not aware of any studies that have evaluated the long-term clinical or process-of-care impacts of pathways related to kidney disease care. Online pathways are a novel approach to disseminate clinical guidelines broadly; they are increasingly being implemented to improve clinical care and, while the observed improvements in this study were small, it is encouraging to note that, at a population-based level, they may have considerable impact on patient care. New strategies to improve CKD identification and management in the primary care setting are particularly important considering that CKD prevalence and incidence have risen considerably (over 85%) in recent decades and, consequently, CKD identification has been recognized as a significant public health priority [48].

Strengths of our study include a population-based cohort with comprehensive health data and the use of an interrupted times series methodology, which has been recommended as a robust approach for clinical pathway evaluation [15]. In addition, the differential uptake of the CKD pathway across the province created the opportunity to use comparison groups to strengthen the analysis. However, there are several limitations that should be acknowledged. First, it was not possible to determine which providers utilized the CKD pathway or which patients were treated according to the CKD pathway. Second, the albuminuria sub-cohort was relatively small, resulting in estimates of change in ACEi/ARB use in that cohort that had wide confidence intervals. Finally, there are limitations inherent in using administrative data – for example, disease diagnoses are based on imperfect algorithms.

## Conclusions

In summary, following implementation of an online CKD clinical pathway, small but statistically significant improvements in ACR testing were evident in the Calgary zone, particularly among individuals without diabetes. Considering these results in combination with the differential uptake of the CKD pathway by geographical zone, the results suggest the CKD pathway may have contributed to increased ACR testing, although the association with other aspects of CKD management or clinical outcomes remains unclear. Targeted dissemination of an online clinical pathway may contribute to improvement in some aspects of medical management of CKD.

## Supplementary Information

**Additional file 1: Table S1**. Characteristics of cohorts used for assessing the secondary outcomes of ACEi/ARB and Statin dispense (p 2-3). **Table S2**. Sensitivity analyses for the primary outcome: estimates of the pre-to-post change in slope for ACR measurements by zone (p 4). **Table S3**. Sensitivity analyses for ACEi/ARB use: estimates of the pre-post change in slope for ACEi/ARB use in a modified quarter, for the diabetes cohort and the cohort with severe albuminuria and no diabetes (p 5). **Table S4**. Sensitivity analyses for statin use: estimates of the pre-post change in slope for statin use in a modified calendar quarter, for the diabetes cohort and the no diabetes/older than 50 cohort (p 6). **Figure S1**. Alberta Health Services Zone Map (p 7). **Figure S2**. Adjusted proportion of patients in the Calgary zone with an ACR measurement in a 28-day period (p 8). **Figure S3**. Adjusted proportion of patients in the Edmonton zone with an ACR measurement in a 28 day period (p 9). **Figure S4**. Adjusted proportion of patients with diabetes who were dispensed an ACEi/ARB in a 28-day period by zone (p 10). **Figure S5**. Adjusted proportion of patients without diabetes but with severe albuminuria who were dispensed an ACEi/ARB in a 28-day period by zone (p 11). **Figure S6**. Adjusted proportion of patients with diabetes who were dispensed a statin in a 28-day period by zone (p 12). **Figure S7**. Adjusted proportion of patients without diabetes but over the age of 50 who were dispensed a statin in a 28-day period by zone (p 13).

## Data Availability

We cannot make our dataset available to other researchers due to our contractual arrangements with the data custodian, the provincial health ministry. Information on how to request a similar dataset is available at https://www.alberta.ca/health-research.aspx.
