# Supplementary material for: Online clinical pathway for chronic kidney disease management in primary care: a retrospective cohort study
Source: BMC Nephrol. 2021 Oct 6;22:332. doi: 10.1186/s12882-021-02533-5 (PMC8496057; doi:10.1186/s12882-021-02533-5)

**Online clinical pathway for chronic kidney disease management in primary care: a retrospective cohort study**

**SUPPLEMENTARY MATERIAL (Contents):**

Supplementary Tables:

- Table S1. Characteristics of cohorts used for assessing the secondary outcomes of ACEi/ARB and Statin dispense (p 2-3).
- Table S2. Sensitivity analyses for the primary outcome: estimates of the pre-to-post change in slope for ACR measurements by zone (p 4).
- Table S3. Sensitivity analyses for ACEi/ARB use: estimates of the pre-post change in slope for ACEi/ARB use in a modified quarter, for the diabetes cohort and the cohort with severe albuminuria and no diabetes (p 5).
- Table S4. Sensitivity analyses for statin use: estimates of the pre-post change in slope for statin use in a modified calendar quarter, for the diabetes cohort and the no diabetes/older than 50 cohort (p 6).

Supplementary Figures:

- Figure S1. Alberta Health Services Zone Map (p 7).
- Figure S2. Adjusted proportion of patients in the Calgary zone with an ACR measurement in a 28-day period (p 8).
- Figure S3. Adjusted proportion of patients in the Edmonton zone with an ACR measurement in a 28 day period (p 9).
- Figure S4. Adjusted proportion of patients with diabetes who were dispensed an ACEi/ARB in a 28-day period by zone (p 10).
- Figure S5. Adjusted proportion of patients without diabetes but with severe albuminuria who were dispensed an ACEi/ARB in a 28-day period by zone (p 11).
- Figure S6. Adjusted proportion of patients with diabetes who were dispensed a statin in a 28-day period by zone (p 12).
- Figure S7. Adjusted proportion of patients without diabetes but over the age of 50 who were dispensed a statin in a 28-day period by zone (p 13).

Table S1. Characteristics of cohorts used for assessing the secondary outcomes of ACEi/ARB and Statin dispense (% unless noted).

|  | Diabetes  (N = 3,414,791 patient records) | Severe albuminuria, no diabetes  (N = 70,897 patient records) | No diabetes, aged 50 years or older  (N = 6,695,062 patient records) |
| --- | --- | --- | --- |
| Number of unique patients | 103,106 | 4,887 | 241,172 |
| Number of times each patient appears in the cohort, median (IQR) | 26 (12, 52) | 13 (6,18) | 20 (11,42) |
| Age, years, median (IQR) | 76.5 (68.8, 83.3) | 68.1 (54.3, 79.9) | 77.6 (68.9, 84.8) |
| Female | 50.6 | 37.3 | 59.5 |
| Zone  Calgary Zone  Edmonton Zone  Other Health Zones | 30.5  32.9  36.6 | 36.3  35.6  28.1 | 36.8  28.5  34.7 |
| Most recent ACR in past year  Normal/mild (A1: <30 mg/g)  Moderate (A2: 30-300 mg/g)  Severe (A3: >300 mg/g)  Unmeasured | 24.2  15.1  8.0  52.7 | 0  0  100  0 | 8.3  2.6  0.9  88.2 |
| eGFR category (ml/min/1.73m^2^)  3a (45-59)  3b (30-44)  4 (15-29)  5 (<15) | 57.3  30.2  11.0  1.5 | 32.3  32.1  27.4  8.2 | 71.2  22.9  5.3  0.6 |
| Number of outpatient serum creatinine measurements in past year  1  2-3  $\geq$4 | 31.1  39.5  29.4 | 15.5  34.1  50.7 | 51.8  33.2  15.0 |
| Alcohol misuse | 4.0 | 5.0 | 2.7 |
| Asthma | 6.2 | 4.3 | 4.1 |
| Atrial fibrillation | 20.1 | 14.5 | 17.0 |
| Cancer | 13.7 | 10.6 | 14.0 |
| Chronic heart failure | 29.1 | 19.0 | 17.8 |
| Chronic pulmonary disease | 31.1 | 23.6 | 24.8 |
| Chronic viral hepatitis B | 0.2 | 0.5 | 0.1 |
| Cirrhosis | 1.1 | 0.9 | 0.5 |
| Dementia | 12.4 | 5.3 | 11.4 |
| Diabetes | 100 | 0 | 0 |
| Epilepsy | 2.4 | 2.2 | 2.3 |
| Hypertension | 94.0 | 87.2 | 77.8 |
| Hypothyroidism | 22.8 | 15.8 | 23.7 |
| Inflammatory bowel disease | 1.6 | 1.8 | 1.9 |
| Irritable bowel syndrome | 3.1 | 1.5 | 3.3 |
| Metastatic cancer | 4.1 | 3.5 | 4.1 |
| Multiple sclerosis | 0.8 | 0.6 | 0.7 |
| Myocardial infarction | 11.5 | 7.2 | 6.9 |
| Parkinson’s disease | 2.4 | 1.0 | 2.1 |
| Peripheral vascular disease | 7.1 | 5.4 | 4.5 |
| Psoriasis | 1.6 | 1.4 | 1.2 |
| Rheumatoid arthritis | 5.7 | 7.0 | 6.1 |
| Schizophrenia | 1.8 | 1.3 | 1.2 |
| Stroke or TIA | 24.6 | 17.3 | 19.4 |
| Neighbourhood income quintile  1 (lowest)  2  3  4  5 (highest)  Unknown | 28.0  23.9  19.6  15.1  13.4  0.1 | 29.4  24.4  18.5  14.5  13.1  0.1 | 24.2  22.4  19.8  16.3  17.1  0.1 |
| Rural residence | 22.9 | 21.7 | 22.1 |

IQR – interquartile range; ACR – urine albumin/creatinine ratio; eGFR – estimated glomerular filtration rate; TIA – transient ischemic attack

Table S2. Sensitivity analyses for the primary outcome (reduced cohorts including only patient records where the mean eGFR was based on 2 or more serum creatinine measurements in the prior year): estimates of the pre-to-post change in slope for ACR measurements by zone.

| Cohort | Patient records | Odds Ratios (per year) for pre-to-post changes in slope | | |
| --- | --- | --- | --- | --- |
|  |  | Calgary Zone | Edmonton Zone | Other Zones |
| Patient records where the mean eGFR was based on 2 or more serum creatinine measurements | 3,911,495 | 1.13 (1.10 – 1.17) | 0.90 (0.87 – 0.93) | 1.01 (0.98 – 1.04) |
| As above, but only patients with diabetes | 1,122,632 | 1.08 (1.04 – 1.13) | 0.93 (0.89 – 0.97) | 1.01 (0.97 – 1.05) |
| As above, but only patients without diabetes | 2,788,863 | 1.16 (1.11 – 1.20) | 0.87 (0.83 – 0.91) | 0.99 (0.95 – 1.04) |

P for interaction between zone and the primary outcome was <.001 in all cases. Adjusted for age, sex, eGFR category, ACR category, the thirteen 28-day periods, neighbourhood income quintile, rural residence, and all comorbidities in Table 1.

Table S3. Sensitivity analyses for ACEi/ARB use: Estimates of the pre-post change in slope for ACEi/ARB use in a modified quarter, for the diabetes cohort and the cohort with severe albuminuria and no diabetes.

|  | Diabetes cohort  (N = 1,080,128 patient records) | | No diabetes, severe albuminuria cohort (N = 22,976 patient records) | |
| --- | --- | --- | --- | --- |
|  | P for interaction | Odds ratio, per year (95% CI) | P for interaction | Odds ratio, per year (95% CI) |
| Overall |  | 1.03 (1.02-1.04) |  | -- |
| Calgary Zone | Ref. |  | Ref. | 0.84 (0.73-0.96) |
| Edmonton Zone | 0.32 |  | 0.01 | 1.07 (0.94-1.22) |
| Other zones | 0.77 |  | 0.10 | 1.00 (0.85-1.17) |

Adjusted for age, sex, eGFR category, ACR category, the thirteen 28-day periods, neighbourhood income quintile, rural residence, and all comorbidities in Table 1.

Table S4. Sensitivity analyses for statin use: Estimates of the pre-post change in slope for statin use in a modified calendar quarter, for the diabetes cohort and the no diabetes/older than 50 cohort.

|  | Diabetes cohort  (N=1,082,126 patient records) | | No diabetes, older than 50 cohort  (N=2,184,098 patient records) | |
| --- | --- | --- | --- | --- |
|  | P value for interaction | OR (95% CI) per year | P value for interaction | OR (95% CI) per year |
| Calgary Zone | Ref | 1.05 (1.03-1.07) | Ref | 1.03 (1.01-1.04) |
| Edmonton Zone | 0.01 | 1.09 (1.07-1.11) | 0.002 | 1.06 (1.05-1.08) |
| Other zones | 0.29 | 1.04 (1.02-1.06) | 0.18 | 1.04 (1.03-1.06) |

Adjusted for age, sex, eGFR category, ACR category, the thirteen 28-day periods, neighbourhood income quintile, rural residence, and all comorbidities in Table 1.

**Supplemental figures**

Figure S1. Alberta Health Services Zone Map


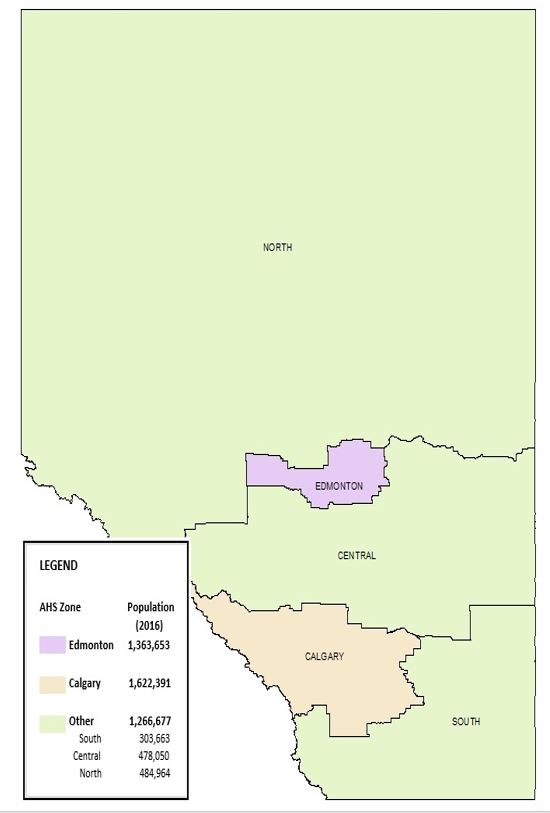


Figure S2. Adjusted proportion of patients in the Calgary zone with an ACR measurement in a 28-day period, from the segmented regression model and a model with period treated categorically. Both models were adjusted for age, sex, eGFR category, neighbourhood income quintile, rural residence, and all comorbidities in Table 1; the segmented regression model was also adjusted for the thirteen 28-day periods and the linear trends.


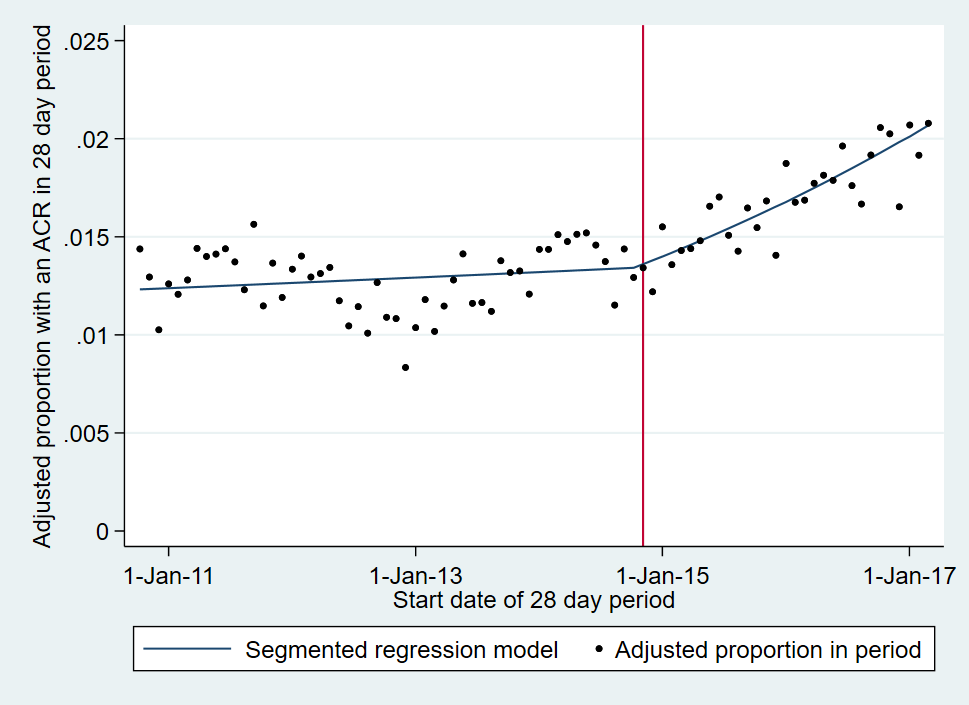


Figure S3. Adjusted proportion of patients in the Edmonton zone with an ACR measurement in a 28-day period, from the segmented regression model and a model with period treated categorically. Both models were adjusted for age, sex, eGFR category, neighbourhood income quintile, rural residence, and all comorbidities in Table 1; the segmented regression model was also adjusted for the thirteen 28-day periods and the linear trends.


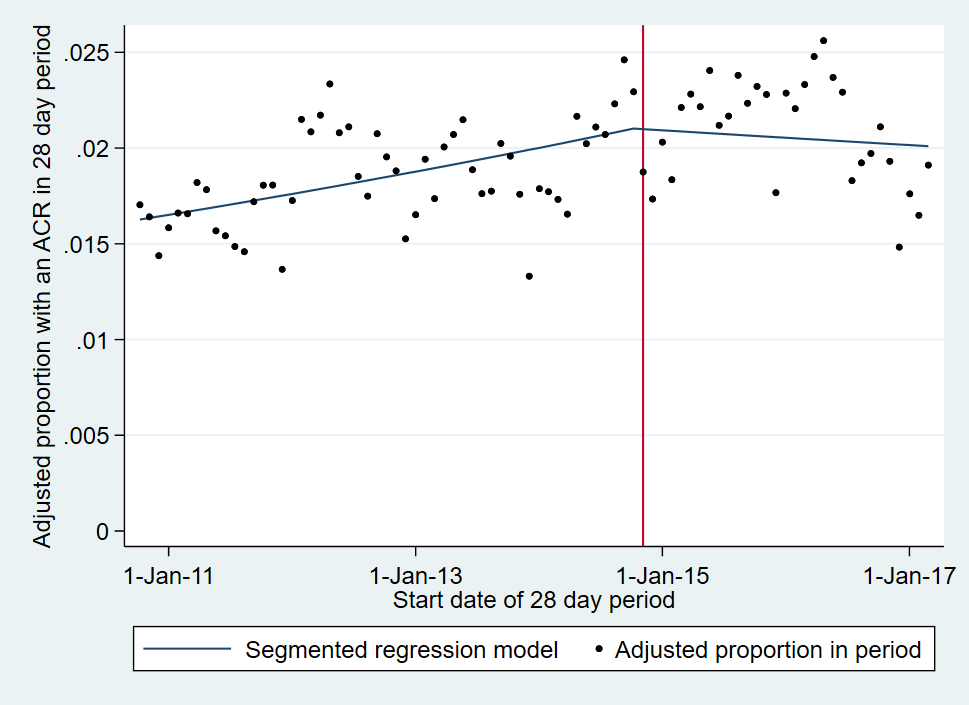


Figure S4. Adjusted proportion of patients with diabetes who were dispensed an ACEi/ARB in a 28-day period by zone, from a segmented regression model. Adjusted for age, sex, eGFR category, ACR category, the thirteen 28-day periods, neighbourhood income quintile, rural residence, and all comorbidities in Table 1.


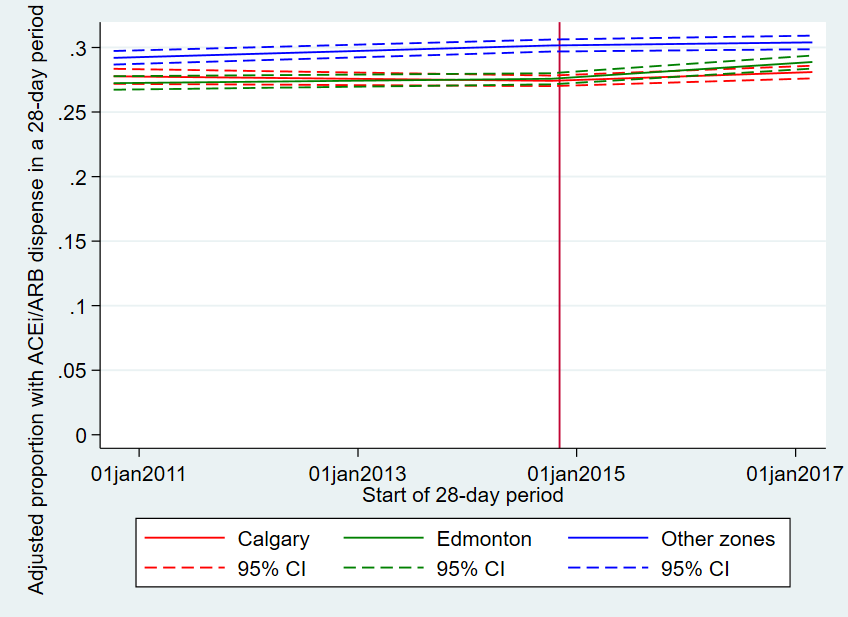


Figure S5. Adjusted proportion of patients without diabetes but with severe albuminuria who were dispensed an ACEi/ARB in a 28-day period by zone, from a segmented regression model. Adjusted for age, sex, eGFR category, the thirteen 28-day periods, neighbourhood income quintile, rural residence, and all comorbidities in Table 1.


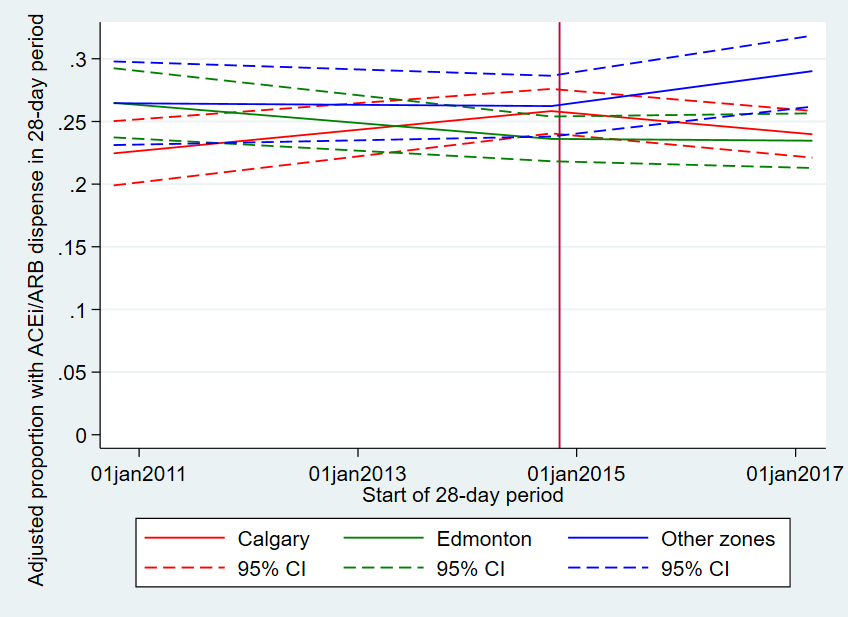


Figure S6. Adjusted proportion of patients with diabetes who were dispensed a statin in a 28-day period by zone, from a segmented regression model. Adjusted for age, sex, eGFR category, ACR category, the thirteen 28-day periods, neighbourhood income quintile, rural residence, and all comorbidities in Table 1.


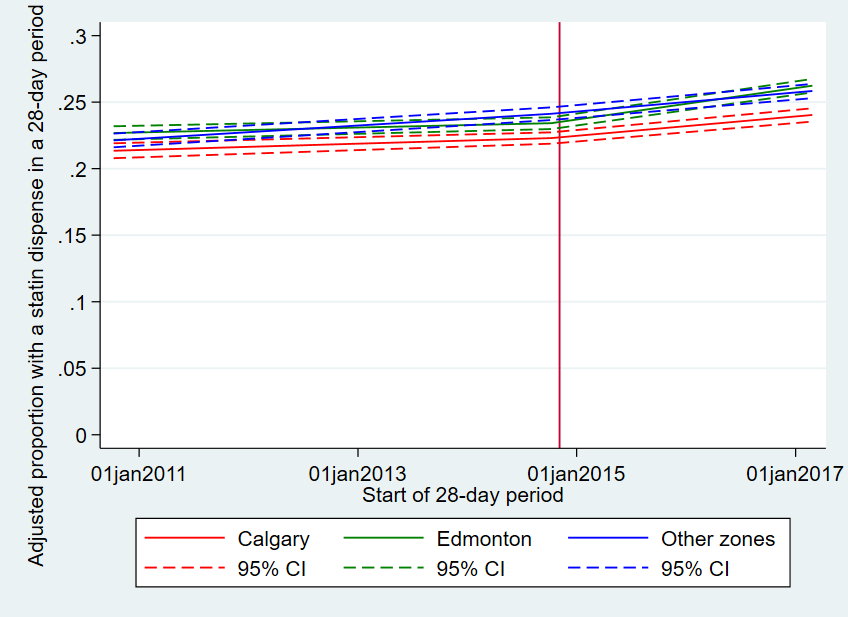


Figure S7. Adjusted proportion of patients without diabetes but over the age of 50 who were dispensed a statin in a 28-day period by zone, from a segmented regression model. Adjusted for age, sex, eGFR category, ACR category, the thirteen 28-day periods, neighbourhood income quintile, rural residence, and all comorbidities in Table 1.


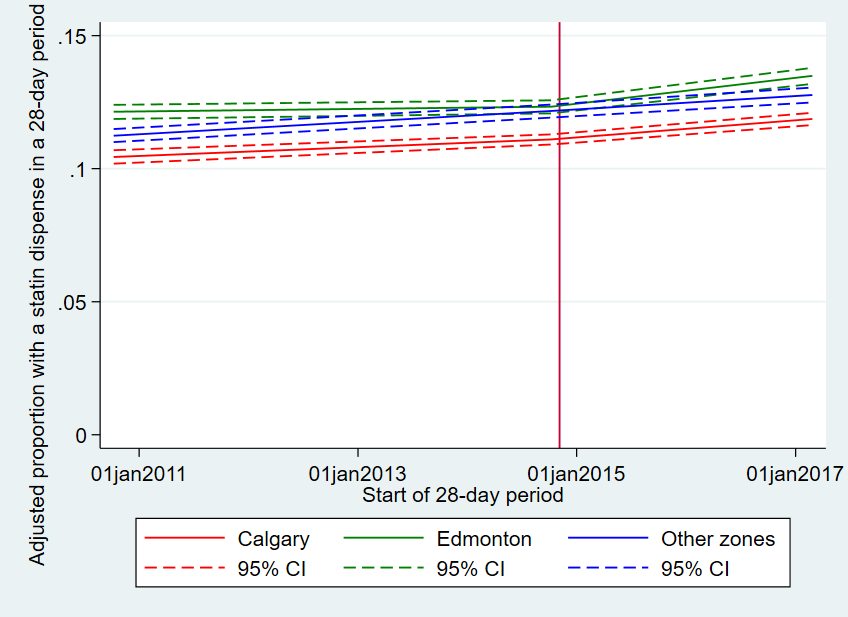

Supplement: Supplementary file 1 — Additional file 1: Table S1. Characteristics of cohorts used for assessing the secondary outcomes of ACEi/ARB and Statin dispense (p 2-3). Table S2. Sensitivity analyses for the primary outcome: estimates of the pre-to-post change in slope for ACR measurements by zone (p 4). Table S3. Sensitivity analyses for ACEi/ARB use: estimates of the pre-post change in slope for ACEi/ARB use in a modified quarter, for the diabetes cohort and the cohort with severe albuminuria and no diabetes (p 5). Table S4. Sensitivity analyses for statin use: estimates of the pre-post change in slope for statin use in a modified calendar quarter, for the diabetes cohort and the no diabetes/older than 50 cohort (p 6). Figure S1. Alberta Health Services Zone Map (p 7). Figure S2. Adjusted proportion of patients in the Calgary zone with an ACR measurement in a 28-day period (p 8). Figure S3. Adjusted proportion of patients in the Edmonton zone with an ACR measurement in a 28 day period (p 9). Figure S4. Adjusted proportion of patients with diabetes who were dispensed an ACEi/ARB in a 28-day period by zone (p 10). Figure S5. Adjusted proportion of patients without diabetes but with severe albuminuria who were dispensed an ACEi/ARB in a 28-day period by zone (p 11). Figure S6. Adjusted proportion of patients with diabetes who were dispensed a statin in a 28-day period by zone (p 12). Figure S7. Adjusted proportion of patients without diabetes but over the age of 50 who were dispensed a statin in a 28-day period by zone (p 13). [file 12882_2021_2533_MOESM1_ESM.docx]
